# The mix and match approach in primary total hip arthroplasty reveals comparable or lower revision rates to matched components: a systematic review

**DOI:** 10.1007/s00402-025-05979-6

**Published:** 2025-07-21

**Authors:** Lukas Leitner, Magdalena Postruznik, Alexander Draschl, Amir Koutp, Andreas Leithner, Patrick Sadoghi

**Affiliations:** 1 Department of Orthopaedics and Trauma Surgery, Musculoskeletal University Center Munich (MUM), LMU University Hospital, Munich, Germany; 2 Department of Orthopedics and Trauma, Medical University of Graz, Graz, Austria

**Keywords:** Total Hip Arthroplasty, Survival Rate, Mix and Match

## Abstract

**Introduction:**

The mix and match (stem and cup from different manufacturers/systems, MM) approach in primary total hip arthroplasty (THA) involves combining components from different manufacturers. Despite various configurations discussed in literature and evidence supporting the safety of MM, controversy persists regarding safety and long term outcomes compared to matched components. Our study aimed to compare the revision rates of MM versus matched components.

**Materials and methods:**

Two databases were searched for English full-text articles published until January, 2024 that evaluated revision rates after primary MM THA. Additionally, MM revision rates data was extracted from the German Arthroplasty Registry (EPRD). The Newcastle-Ottawa Scale (NOS) for cohort studies was used for quality assessment.

**Results:**

Three national and one hospital registry studies were included, of which three demonstrate MM as a common practice (19–24%). All studies found comparable revision rates for MM cohorts, or even slightly improved survival rates in MM cohorts concerning revision rate and PROMs, mostly lacking clinical relevance. These findings align with the data reported in the EPRD, with revision rates of approximately 3.6% after 6 years in both MM and matched THA.

**Conclusions:**

Employing MM in primary THA presents a feasible and safe approach, capable of providing custom fit tailored to individual patients with revision rates comparable to those of matched THA.

**Supplementary Information:**

The online version contains supplementary material available at 10.1007/s00402-025-05979-6.

## Introduction

The implantation of components from different manufacturers (referred to as the mix and match (MM) approach) in total hip arthroplasty (THA) has gained considerable attention owing to its potential for customized solutions, cost-effectiveness, flexibility, and optimized resource management [16]. Having gained acceptance in revision surgery and progressing alongside orthopedic innovations, this approach allows implants to be tailored to individual patient needs and surgeons’ routine [10]. However, obstacles encompass compatibility concerns, adherence to regulatory standards, and the limited availability of comparable data [8].

While the compatibility of components within a single manufacturing facility undergoes prior testing [2], the compatibility of components sourced from different manufacturers remains unverified in terms of prior evaluation [14]. Therefore, mixing components from different manufacturers leads to its categorization as an off-label use, which can result in legal issues, in case of implant failure, in several countries [10]. This entails the application of medical products, such as joint implant components, in manners not approved by regulatory agencies or the manufacturers themselves. In the past, several authors categorically advised against MM use in primary THA [3, 6]. Presently, mixing components in primary THA has still emerged as an important topic due to its potential advantages. Surgeons are increasingly exploring this approach to tailor implants according to the distinct requirements of individual patients, control costs, and optimize surgical procedures while ensuring compatibility and regulatory adherence. Application of MM seems quite common in some regions, and in fact, MM in THA has been practiced on a large scale according to a recent EFORT recommendation commentary on MM [15].

Typically, modern state of the art mixing entails employing a cup and liner of one company combined with a stem and head sourced from a different manufacturer. This combination usually involves pairing a polythene (PE) inlay with ceramic or metal heads [14]. Use of hard-on-soft bearings is recommended in case of MM in different manufacturers, since polyethylene liners are requested to be slightly larger than bearing size by standardization (ISO 7206-2), reducing any probability of pinching, when head and liner from different manufacturers are combined [15]. However, diverse mixing methods are documented in the existing literature.

Solely the combination of femoral stems and femoral heads from different companies should clearly be avoided at the moment: Even when carrying identical labels, tapers may not achieve proper compatibility with the femoral head because of the prevailing absence of standardization [7, 8]. In light of this non-standardization, several authors and case reports advised against mixing a stem and a head from different manufacturers [4, 5, 8].

In summary, mix-and-match approaches are already widely practiced. However, due to the heterogeneity of the existing literature and the still limited evidence from long-term results, this type of treatment continues to pose a legal issue in cases of failure. With this backdrop, our review sets its focus on the revision rates in primary THA within the context of the MM versus matched approach, delving into a systematic review of recent comparable research findings, with focus on long-term results.

## Materials and methods

### Identification and selection of trials

In January 2024, two independent reviewers (A.D. and M.P.) conducted a systematic search of the literature adhering to the Preferred Reporting Items for Systematic Reviews and Meta-Analyses (PRISMA) guideline using the PubMed and EMBASE databases. The objective was to identify articles reporting revision rates in primary total hip arthroplasty (THA) employing the MM and matched approach. The literature search was limited to results published until January 2024.

The search strategy involved the following search string for both databases: ((total hip arthroplasty) AND (mix)) AND (match) as well as (total hip arthroplasty) AND (different manufacturer). In addition, references cited by the included articles were sought for potential inclusion. During that process, there was no disagreement between the two reviewers, hence consultation with the senior author (P.S.) was not necessary.

**Fig. 1:**
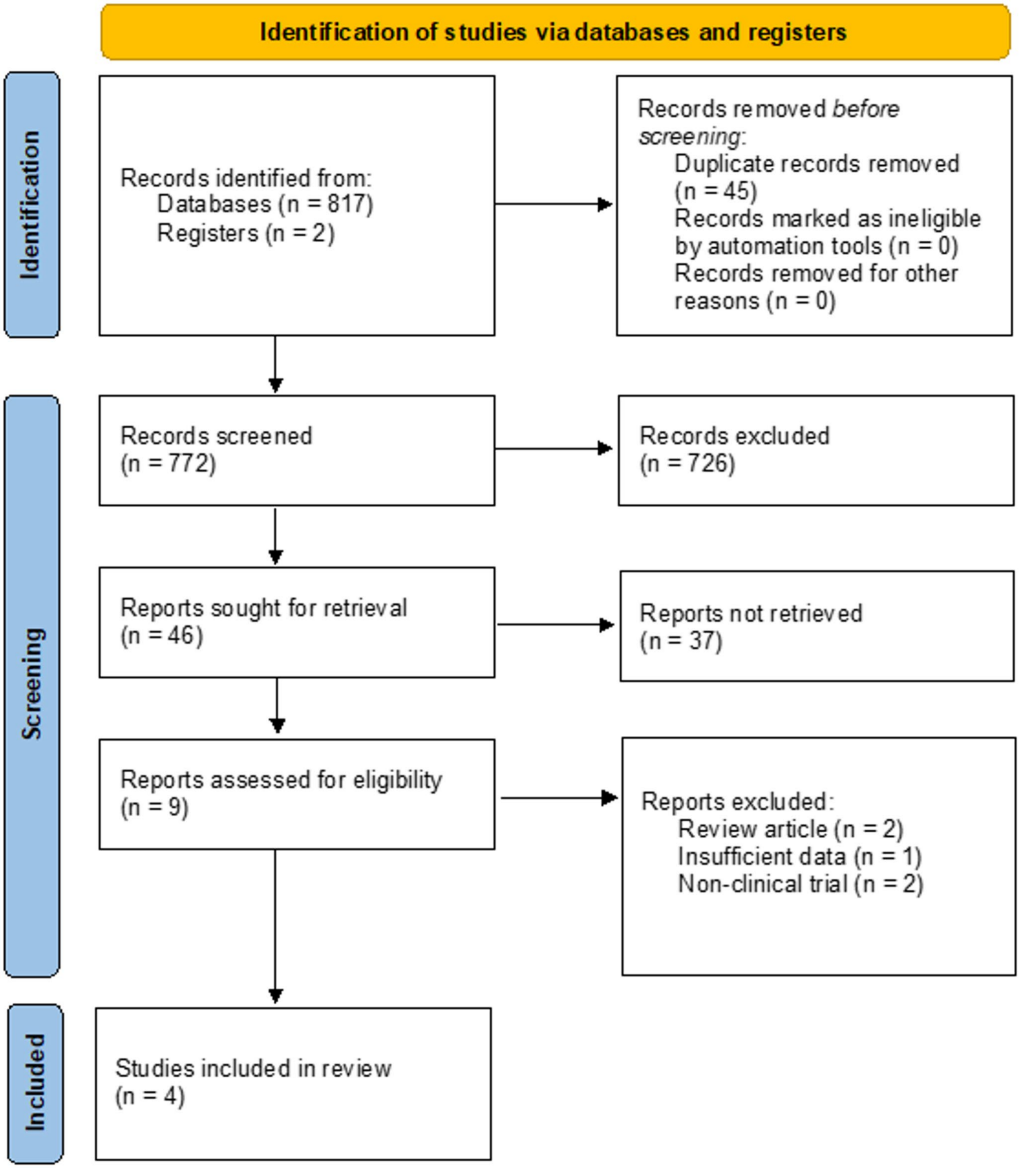
PRISMA THA study flow diagram presenting the selection process, revealing four studies for final analysis. *PRISMA* Preferred Reporting Items for Systematic Reviews and Meta-Analyses, * THA* total hip arthroplasty

The reviewers evaluated the articles for eligibility through screening of the title and abstract first, followed by full text assessment (Fig. 1). Within the process of identification, no automation tool was used to mark articles as ineligible. Inconsistencies in agreement were resolved by consensus and the senior author (P.S.) was involved if there was disagreement.

### Eligibility criteria

Studies were considered eligible for qualitative analysis if they met the following criteria: (1) comparison of adult patients who underwent primary MM and matched THA; (2) a follow-up of at least 6 years; (3) revision rates either deducible from the available dataset or explicitly mentioned in the manuscript; (4) published in a peer-reviewed journal and written in English or German language; and (5) the scope of eligibility extended solely to clinical trials and registry studies. Exclusion criteria were case reports, reviews and meta-analyses, studies involving cadaveric or animal subjects, investigations focused on pharmaceutical interventions, prospective studies, and articles including pediatric populations.

### Data collection process and data items

Data collection was carried out directly from the included articles into an Excel sheet extracting the following information: title, year of publication, origin of the corresponding author, publishing journal, sample size, fixation type, follow-up in years, and annual re-vision rates. This process was also done by two independent researchers (A.D. and M.P.) with any disagreements either resolved by consensus or decision of the senior author (P.S.). Any missing information in the studies is denoted “NR” (not reported) in the corresponding tables. Data from the German Arthroplasty Registry (EPRD) were collected from the annual report from 2023 [1].

### Risk of Bias assessment and certainty of evidence (Quality assessment)

The quality of the included studies was assessed by the same pair of independent re-viewers who were engaged in the search process (A.D. and M.P.) using the Methodological Index for Non-Randomized Studies (MINORS), with scores ranging from 0 to 16 and from 0 to 24 for non-comparative and comparative studies, respectively; higher scores reflect higher quality [12]. For this review a score of ≤ 14 was considered to be poor quality, 15–22 moderate quality, and 23–24 good quality for comparative studies. In the event of discrepancies between the two reviewers, they were resolved by consensus or by decision of the senior author (P.S.).

A Level of Evidence assessment was conducted according to the Oxford Centre for Evidence-Based Medicine recommendations from 2011.

### Data synthesis and analysis (outcome measures)

The primary outcome measures in this review were the cumulative revision rates of MM and matched THA with a follow-up of at least six years after primary replacement. Given the limited number of included studies, quantitative analysis was not possible.

## Results

### Flow of trials through the review

The literature search identified 772 potentially relevant studies. After title and abstract screening, fulltext screening was performed on nine remaining studies. Of those, two review articles [15, 18] and two non-clinical studies were excluded. Additionally, one study [17] was excluded due to insufficient description concerning the methodology. Consequently, four studies [11, 13, 14, 16] met the eligibility criteria and were included in the final qualitative analysis. Among the included studies, one used the arthroplasty registry of a single Slovenian hospital [14], and the remaining three used national arthroplasty registries from England and Wales [16], New Zealand [13], and the Netherlands [11].

### Participants and THA approach

Overall, the studies comprised 710,077 patients who underwent primary THA, of which 18.2% (*n* = 128,983) underwent THA via the mix-and-match approach. Further details of the studies’ demographic data are listed in Table 1.

**Table 1:** Evaluation of the included trials

	Tucker et al. [16]	Peters et al. [11]	Taylor et al. [13]	Trebše et al. [14]
Register	National Joint Registry for England and Wales	Dutch Arthroplasty Register (LROI)	New Zealand Joint Registry (NZJR)	Valdotra Orthopaedic Hospital Arthroplasty Registry
Country	United Kingdom	The Netherlands	New Zealand	Slovenia
Time of surgery	2003–2013	2007–2014	1999–2015	2002–2004
Maximum follow-up	9 years	8 years	17 years	13 years
MINORS^1^	16	15	15	16
Level of Evidence	IV	IV	III	III
Total THA cases (n)	447,058	161,360	99,732	1,927
- Matched THA	81.0%	88.6%	75.4%	44.6%
- Mixed THA	19.0%	11.4%	24.6%	55.4%

### Follow-Up

The maximum follow-up ranged from 8 to 17 years across all studies. Two studies reported the mean [14] and median [11] length of follow-up, whereas the other half of studies have not provided any detailed information regarding median or mean time of follow-up.

### Methodological quality-assessment

All studies were registry studies within a retrospective/prospective cohort study design. The evaluation of study quality revealed that all included studies were of moderate quality according to the MINORS criteria.

### Revision rates

A retrospective study from Slovenia [14] compared 14-year survival of MM (*n* = 1067) and matched prostheses (*n* = 860) implanted from 2002 to 2004, reporting superior survival probability in the matched group compared to the MM group: 96.0% and 92.7% at 14 years, respectively (*p* = 0.002) (Supplementary Table 1). They additionally divided the MM group into two subgroups, depending on whether the stem-head or the stem-cup pairing originated from the same manufacturer. When comparing the subgroup where the stem and head components were from the same manufacturer with the traditionally matched group, there was no significant difference in the survival rate, compared to the matched group, any more (*p* = 0.079). Of note, this was the only, and smallest MM and matched THA registry data available, where a statistically significant inferiority of MM could be detected from the raw data (before adjustment).

Results from a register study conducted by the National Joint Registry of England and Wales (NJR), including 90,000 MM cases [16]. Implant sets were divided into 5 subgroups (Supplementary Table 2). Concerning hard-on-soft bearings, this study even found significant lower revision rates in the MM group (different stem and cup, *n* = 48,156) compared to the matched groups when a cemented modular stem or a monobloc stem was used in combination with a polyethylene cemented cup, resulting in a 8-year cumulative revision percentage of 1.9% (95% CI 1.7–2.1) and 2.4% (95% CI 2.3–2.5), respectively (*p* = 0.001). The results were calculated under assumption of estimated revisions using Kaplan-Meier curves and the curves for the subgroups were compared using log-rank tests. Of note, the revision rates in a small subgroup (*n* = 527) mixing metal heads from one manufacturer with a stem from another manufacturer was associated with a significantly elevated risk of revision than the matched combinations (*p* = 0.001). The authors identified earlier described mechanical corrosion at the taper junction as possible mechanism for this finding [9, 16].

Another registry study from the New Zealand Joint Registry (NZJR) recorded a total of 108,613 primary THAs with a MM cohort of 24,537 (24.6%) THAs with up to 17 years of follow-up [13] (Supplementary Table 3). Data revealed only slightly superior, but statistically significant (*p* = 0.049), survival rates of 4.4% (0.69/100 component years) in the MM group, compared to the matched group (4.6%; 0.72/ 100 component years). This difference vanished, after metal-on-metal or ceramic-on-metal bearings, which were overrepresented in the matched group, were excluded. There was also a small, statistically significant but not clinically relevant improvement in Oxford Hip Scores for the unmatched (score 41.1) group compared to the matched group (score 40.3) in this cohort. The effect of mixing heads and stems from different manufacturers was not analyzed in this study as it is very uncommon in the data from the NZJR (< 0.9%).

Another study, conducted in the Netherlands [11], using data from the nationwide population-based arthroplasty register known as the Dutch Arthroplasty Register (LROI). The study focused on primary total hip arthroplasty (THA) procedures performed between 2007 and 2014, totaling 163,360 cases. The researchers categorized the THAs into four groups based on the manufacturers of the components used (Supplementary Table 4). The groups were as follows: (1) Non-mixed THAs: All components (femoral stem, head, and cup) from the same manufacturer (*n* = 142,964); (2) Mixed stem-head THAs: Different manufacturers for the femoral stem and head (*n* = 3,663); (3) Mixed head-cup: Different manufacturers for the head and cup components (*n* = 12,960); (4) Mixed stem-head-cup THAs: Different manufacturers for the femoral stem, head, and cup components (*n* = 1,773). The study found that MM components were used in approximately 11% of all THAs (*n* = 18,396) over the 8-year period. The 6-year revision rates for both MM and matched THAs were similar, with 3.4% for MM THAs and 3.5% for matched THAs. Whilst the incidence of revision due to loosening of the acetabulum (the cup component) was higher in MM THAs, accounting for 16% of revisions compared to 12% in matched THAs (16% vs. 12% in non-mixed THAs; *p* < 0.05), symptomatic metal-on-metal revisions were rarer in MM THAs (1.7% vs. 6.6%; *p* < 0.001), but only due to the higher prevalence of metal-on-metal cases in matched THAs. Overall, the interpretation of the study’s results suggests that over the 8-year period in the Netherlands, a significant proportion of THAs (11%) involved the use of mixed components and the medium-term revision rates were comparable between MM and matched THAs. Of note, the authors suggest the comparison of pooled international data and longer follow-up data for future analysis.

Additionally, MM revision rates data was extracted from the German Arthroplasty Registry (EPRD) [1], revealing a 6-year revision rate for matched primary THR of 3,61% (8919 revisions in 246,885 cases) compared to 3,58% in MM cases (337/9,398).

Therefore, our analysis within this review indicates that initial one-year and six-year revision rates for MM and matched THAs are comparable throughout published registry data studies. From these selected data, revision rates even slightly favored mixed stem-cup components initially in the first years but did not differ significantly after 6 years, with the important limitation, that stem and femoral head must be from the same manufacturer.

## Discussion

There is an ongoing debate about the safety of MM approaches in primary THA in the orthopedic community, represented by a high proportion of MM primary THA (19–24%) in this current analysis on 4 European and the New Zealand Registries. This is partly due to the fact that current literature and guidelines do not provide clear and definitive recommendations on the matter. In contrast to e.g. partial revision THA, where mix-and-match approaches have been described as a viable option by EFORT in 2011, provided there is a benefit for the patient [15]. Previous studies have also criticized the lack of international registry data and long-term results in this context [11].

The purpose of this systematic study was to evaluate international registry data using comparable definitions of MM and follow-up data of at least 6 years for the first time, in order to provide new and more reliable evidence on the safety of MM as a method in clinical practice. First, we could show, that MM in primary THA is already a quite commonly used approached besides traditional matched THA, according to several European registries. This is in line with data from a recent EFORT recommendation commentary on MM [15].

Our analysis further demonstrated that MM in THA appears to be a safe method, showing at least similar revision rates compared to matched THA. It is noteworthy that revision rates following MM were in some cases even significantly better [13]. However, this seems to be attributable to the less frequent use of developments such as metal-on-metal bearing surfaces and does not appear to be clinically relevant due to the effect size.

What is undisputed, however, is that combining components such as the stem and femoral head from different manufacturers should be avoided, as all registry data investigating this issue have reported higher revision rates. This problem has also been addressed in earlier studies, and can be described as mismatch in many cases [4, 10]. Furthermore, hard-on-soft bearings should be used in cases of MM combination of head and bearing from different manufacturers, in order to avoid pinching [15].

A legal challenge remains, as MM continues to represent an off-label use, leaving the surgeon/healthcare provider potentially liable to legal action in the event of implant failure. Nonetheless, reference should be made to a study by Peters et al. (2020), which found no legal cases against orthopedic surgeons in this context in the United Kingdom, Germany, and the Netherlands [10]. Studies like ours aim to strengthen the legal framework surrounding the safe application of MM, which is already widely practiced, by providing further evidence and support on the safety of this technique.

It is still recommended to inform patients about the use of MM prior to surgery, preferably in writing, and to document this consent formally. In the future, a position statement from national and international orthopedic societies, establishing expert consensus to legitimize this practice, should be sought. Additionally, it would be beneficial to advocate for expanded compatibility data provided by implant manufacturers (who are, by nature, not particularly interested in certifying their products for use with those of other manufacturers). It also further remains necessary for the surgeon to have profound knowledge with respect to interactions between different materials of different implants and what combinations are feasible.

### Study limitations

The issue of data heterogeneity and the variability of materials used is explicitly emphasized in the interpretation of the results.

## Conclusion

Analysis of 4 European and the New Zealand THA registries revealed that use of MM THA demonstrates similar implant survival rates to matched THA over an observation period of at least six years, with indications that this may also apply to long-term outcomes. The recommendation to avoid combining femoral heads and stems from different manufacturers remains valid, as this often results in a mismatch and leads to higher failure rates.

## Supplementary Information

Below is the link to the electronic supplementary material.

Supplementary file1 (DOCX 34 KB)

## Data Availability

No datasets were generated or analysed during the current study.
