# Supplementary material for: The mix and match approach in primary total hip arthroplasty reveals comparable or lower revision rates to matched components: a systematic review
Source: Arch Orthop Trauma Surg. 2025 Jul 21;145(1):381. doi: 10.1007/s00402-025-05979-6 (PMC12279603; doi:10.1007/s00402-025-05979-6)
Supplement: Supplementary file 1 — Supplementary file1 (DOCX 34 KB) [file 402_2025_5979_MOESM1_ESM.docx]

**Supplementary Table 1.** Revision rates of THA in the different subgroups of the Slovenian single hospital register study [14].

**Supplementary Table 2.** Revision rates of THA in the different subgroups of the NJR register study [16].

**Supplementary Table 3.** Revision rates of THA in the different subgroups of the NZJR register study [13].

**Supplementary Table 4.** Revision rates of THA in the different subgroups of the LROI register study [11].

**Supplementary Table 1**

| **Years after surgery** | **Revision rates of different THA approaches (%)** | | |
| --- | --- | --- | --- |
|  | non-mixed  (n=860) | mixed stem-head THA  (n=926) | mixed head-cup THA  (n=141) |
| 1 | 0.6 | 0.8 | 0.1 |
| 2 | 0.6 | 1.2 | 0.1 |
| 3 | 0.9 | 1.5 | 0.1 |
| 4 | 0.9 | 2.0 | 1.4 |
| 5 | 1.2 | 2.5 | 1.4 |
| 6 | 1.5 | 3.0 | 2.2 |
| 7 | 1.7 | 3.7 | 2.2 |
| 8 | 2.0 | 4.8 | 3.0 |
| 9 | 2.3 | 5.2 | 3.8 |
| 10 | 2.8 | 6.0 | 6.3 |
| 11 | 3.2 | 6.5 | 7.4 |
| 12 | 3.5 | 6.7 | 7.4 |
| 13 | 4.0 | 7.4 | 7.4 |

**Supplementary Table 2**

| **Subgroup** | **Approach** | **Stem fixation** | **Cases (n)** | **Revisions (n)**  **after 8 years** | **Revision (%)**  **after 8 years** |
| --- | --- | --- | --- | --- | --- |
| 1^a^ | Matched | Cemented | 158178 | 2155 | 1.4% |
| 1^a^ | Different stem and cup | Cemented | 48156 | 586 | 1.2% |
| 2^b^ | Matched | Cemented | 35558 | 469 | 1.3% |
| 2^b^ | Different cup | Cemented | 24004 | 386 | 1.6% |
| 2^b^ | Different stem | Cemented | 136 | 2 | 1.5% |
| 2^b^ | Stem, head, and cup mixed | Cemented | 439 | 11 | 2.5% |
| 2^b^ | Matched | Uncemented | 72742 | 1386 | 1.9% |
| 2^b^ | Different cup | Uncemented | 5906 | 107 | 1.8% |
| 2^b^ | Different stem | Uncemented | 317 | 5 | 1.6% |
| 2^b^ | Stem, head, and cup mixed | Uncemented | 152 | 3 | 2.0% |
| 3^c^ | Matched | Cemented | 14836 | 192 | 1.3% |
| 3^c^ | Different head and cup | Cemented | 1209 | 5 | 0.4% |
| 3^c^ | Matched | Uncemented | 68459 | 1250 | 1.8% |
| 3^c^ | Different head and cup | Uncemented | 2652 | 41 | 1.5% |
| 4^d^ | Matched | Cemented | 952 | 87 | 9.1% |
| 4^d^ | Different cup | Cemented | 1 | 0 | 0.0% |
| 4^d^ | Different stem | Cemented | 514 | 75 | 14.6% |
| 4^d^ | Stem, head, and cup mixed | Cemented | 0 | 0 | 0.0% |
| 4^d^ | Matched | Uncemented | 11350 | 1507 | 13.3% |
| 4^d^ | Different cup | Uncemented | 0 | 0 | / |
| 4^d^ | Different stem | Uncemented | 1492 | 109 | 7.3% |
| 4^d^ | Stem, head, and cup mixed | Uncemented | 5 | 2 | 40.0% |

^a^ Modular stem or monobloc system with ceramic or metal heads and PE cemented cup

^b^ Modular stems with a metal head and a metal shell with PE liner

^c^ Modular stems with a ceramic head and a metal shell with ceramic liner

^d^ Modular stems with metal heads used with a metal monobloc cup designed for hip resurfacing

**Supplementary Table 3**

| **Years after surgery** | **Revision rates of different THA approaches (%)** | |
| --- | --- | --- |
|  | non-mixed  (n=75,195) | mixed stem-cup THA  (n=24,537) |
| 1 | 1.1 | 1.1 |
| 2 | 1.6 | 1.5 |
| 3 | 2.3 | 1.9 |
| 4 | 2.8 | 2.3 |
| 5 | 3.3 | 2.6 |
| 6 | 3.9 | 3.1 |
| 7 | 4.5 | 3.8 |
| 8 | 5.2 | 4.4 |
| 9 | 5.9 | 5.0 |
| 10 | 6.7 | 6.0 |
| 11 | 7.6 | 6.9 |
| 12 | 8.9 | 8.0 |
| 13 | 10.0 | 9.3 |
| 14 | 11.5 | 10.8 |
| 15 | 12.7 | 12.5 |
| 16 | 14.0 | 14.7 |
| 17 | 15.6 | 16.2 |

**Supplementary Table 4**

| **Years after surgery** | **Revision rates of different THA approaches (%)** | | | |
| --- | --- | --- | --- | --- |
|  | non-mixed  (n=142,964) | mixed stem-head THA  (n=3663) | mixed head-cup THA  (n=12,960) | all mixed THA  (n=1,773) |
| 1 | 1.3 | 0.8 | 1.5 | 1.2 |
| 2 | 1.9 | 1.2 | 2.1 | 1.7 |
| 3 | 2.4 | 1.6 | 2.4 | 2.5 |
| 4 | 2.8 | 2.1 | 2.8 | 3.0 |
| 5 | 3.1 | 2.6 | 3.2 | 3.4 |
| 6 | 3.5 | 3.2 | 3.4 | 3.5 |
